# Book Reviews

**Published:** 1806-08

**Authors:** 


### CRITICAL ANALYSIS
OF THE
### RECENT PUBLICATIONS
ON THE
### DIFFERENT BRANCHES OF PHYSIC, SURGERY, AND MEDICAL PHILOSOPHY.

---

### *The Edinburgh Medical and Surgical Journal,* Number 7. July 1806.

Article 1.—*Observations on the Structure of the Parts concerned in Crural Hernia.* By ALLAN BURNS, Member of the Royal College of Surgeons in London, and Lecturer on Anatomy and Surgery in Glasgow.

THIS is a long paper, illustrated with two engravings; but engravings, to be useful for any intricacies of parts subject to surgical operation, should not only be as near to the size of life as possible, but coloured also. The eye never can follow, with any satisfaction, a mere black and white representation, which, for practical purposes, must be useless. As to the remarks, we give our author credit for some minutiæ which he describes; but as subjects of this kind can only be demonstrated on the recent subject, we shall leave the whole in the hands of the anatomical teachers, who doubtless will pay a proper regard to Mr. Burns's industry.

Article 2.—*Observations on the State of the Venereal Disease, by* JOHN WILSON, Surgeon to his Majesty's ship Porpoise.

THIS paper abounds with good sense, well directed, and accurately expressed. It will astonish some of our readers to find, that on the arrival of the Porpoise at Otaheite from port Jackson, Mr. Wilson should not be able to find a single case of well-established lues venerea in the island; and that there was no sufficient proof that any of the men were infected during their stay. The author's remarks are so judicious, that we cannot fail to give them in his own words.

"The total exemption of the inhabitants from lues venerea, and the unfrequency of gonorrhœa, both so irreconcileable with the accounts in voyages, and more especially with the assertions of the missionaries, could not fail to excite surprise, and, at the same time, an attempt on my part to investigate the merits of opinions so very repugnant to our experience; and the result of my inquiry proved, that the missionaries at least had adopted erroneous opinions respecting the nature of the many tumours and ulcers which are to be met with among the inhabitants; for they did not hesitate to to pronounce, that almost every tumour or ulcer, which came under their observation, had its origin in a venereal source.

"Their mistake originated partly from prejudice, and partly from being unqualified to discriminate; for they arrived at this island with as thorough a conviction of the prevalency of lues venerea amongst the inhabitants as we did, and it cannot be supposed that any of them have much medical knowledge. At the same time it must be confessed, that the accounts of the natives often tended to confirm them in this error; for, from a kind of vain credulity, they believe, or rather pretend, that their island, before their intercourse with strangers, was free from most of the diseases to which they are now subject; and thus lues venerea being, on all hands, allowed to be a foreign distemper, they of course most commonly ascribe their sores to that source."

The author afterwards quote several passages from Captain Cook and others, and concludes with the following remarks:

"From the foregoing statement it may be concluded, I hope, without incurring the censure of presuming too much, that, notwithstanding the melancholy accounts we read of the ravages of lues venerea at Otaheite, and even disputations about its first importers, this disease was not introduced there antecedent to the Porpoise's voyage; and that Captain Cook and others met with gonorrhœa only, which they supposed sufficient to produce all the symptoms of pox; and also that it must be sufficiently obvious that the nature of these infections is different, as we found the one prevailing, yet in no instance met with the other.

"However, although the first navigators are thus freed from having introduced this scourge of mankind, it is melancholy to reflect that its speedy importation was almost certain; for, before we departed from these islands, a small vessel arrived from Port Jackson, on board of which there was a man affected with bubo and chancres; and it is probable that the efforts of the master to prevent him from disseminating the infection would prove unavailing.

"Yet there remained a still more certain source of infection from two vessels which afterwards arrived from the Sandwich Islands, where, I have good authority for saying, the disease is very common. And whenever it is imported, there can be little doubt of its making a rapid progress, notwithstanding the benevolent efforts of the missionaries; for, from the danger, and, indeed I fear, the impossibility of making the natives submit to a mercurial course, it will be a very difficult matter to cure any of them.

"The great probability of their future intercourse with New South Wales becoming frequent, will also be a constant source of infection, and bids fair to destroy the intentions of the mission; for from that settlement they have already received, and will continue to receive, deserters, whose baneful example and instructions will not fail to counteract the benefit which would otherwise accrue to these courteous islanders from its exertions.

Article

Article 3.—*Remarks on the Depopulation of Otaheite and Eimeo, with an Account of some of the most common Diseases.* By JOHN WILSON, Surgeon to his Majesty's ship Porpoise.

THIS paper is no way inferior to the last, abounding with the same marks of sound judgment, the same industrious and unprejudiced spirit of research.

The first enquiry is into the probability of a greater population formerly. This is satisfactorily proved. Among the diseases, fever is the most common, beginning with symptoms of the intermittent, and proceeding to remission, under which the patient often continues till nature is exhausted, or the disease produces some local effect which relieves the constitution. As far as we can collect, the sufferers are the poorer sort, who sleep on the ground, from want of better food are less able to resist miasmata; and, when ill, are unable to procure those remedies and those comforts which may cure, or enable them to struggle through the disease. The principal remedy is a removal to the islands a few miles north. The author expresses a doubt whether the fever should be considered as contagious. From his account it seems probable, that, like many other diseases, though not originally contagious, yet among the crowded sick it generates a contagious fever partaking of its own type.

Dysentry is another very fatal disease among them. It raged with great violence whilst the Porpoise was at the island, but the author does not inform us whether it infected any of the crew. Probably it may become contagious only by the same means as their fevers.

Phthisis pulmonalis is more frequent, and becomes fatal sooner than with us.

Rheumatism is also not uncommon, and scrofulous tumours more frequent than in Britain. The author, however, makes allowances for the dress discovering every part affected, whilst in this country only the face, and sometimes the neck are discoverable, by being exposed.

Epilepsy and hysterics are not uncommon, and are considered as demoniacal possessions, or the effect of supernatural intercourse, during which the patients are supposed to be inspired. In consequence of this, some of the older women are very expert at working themselves into, or counterfeiting, hysterics. Some ingenious reflections are added on this subject.

The author concludes with the following very pertinent remarks.

"Some of the diseases which at present ravage these islands may, by some, be supposed to have been imported; but of this we can have no evidence; and probably all the elucidation the subject will admit of may be found in the analogy of many countries which have been long healthy and populous, suddenly becoming overwhelmed with disease and death, from some revolution in nature, which is likely always to remain a mystery.

"Although

" Although disease must have had a very powerful influence on the depopulation, yet the greatest share is to be attributed to a custom which, although it has been practised as a system by others, perhaps more civilized people, is of a nature the most barbarous and cruel that ever disgraced the species. This is infant murder, which is carried to such an extent, that some of the best informed of the missionaries have asserted, that at least two-thirds of the whole of the births on the island are destroyed the moment they have seen the light. Not only is the offspring of an unequal connexion with respect to rank, either on the male or female side, invariably destroyed, but young men and women, who are every way equal, frequently agree to murder the innocent fruit of their pleasures ; and more females are destroyed than males.

" They appear to have no idea of any criminality being attached to this deed, and many of the women who had borne children, on being requested, did not scruple or appear abashed to tell us the number they had destroyed. The young king's wife, although not twenty years of age, has, I think, killed two, which she pretended, were begot by some of her domestic attendants.

" I have remarked, that at least two-thirds of the whole of the women on Otaheite are either middle aged or old, and that children are principally observed to be possessed by the former : for it is a very rare occurrence, indeed, to meet with a young woman rearing a child : likewise, that the whole of the females do not amount to above one-tenth part of the males ; from which it may be inferred, that they do not often save their children until about or after their meridian, and that infant murder has been of late years more frequent than at the time when those middle aged and old women were born. The more frequent destruction of females sufficiently accounts for their disparity to the males. It is a mere question of course with the natives, on hearing the news of a birth, to ask whether it is preserved.

" They have many absurd customs in their treatment of children, particularly females, which certainly render the rearing of them exceedingly troublesome ; but there is nothing of a sacrificial nature in their destruction : for, although they do not suppose that they incur their displeasure by it, yet they do not pretend that it is acceptable to any of their divinities: and, indeed, although they have some notion of a future state, it does not seem to have any influence whatever on their moral actions in this world.

" I am of opinion that infant-murder is more frequently practised since their connection with us, and that it will continue to increase in proportion to the number of ships which may for the future visit them, principally from the following considerations:

" *First*, From all the information which I could procure, I believe their only inducement to this cruel deed, independent of inequality of rank, or being an Arreoyie, which are always insurmountable, and from the construction of their succession, are love love of pleasure and avarice; and these passions, but more particularly the latter, have gained much ground since their intercourse with us: indeed, I have often been struck with astonishment, when observing the eagerness with which they hoard European manufactures. They are also as well aware as we are, that the charms of women who rear families sooner decay; and having experienced that female beauty is always a marketable article with their visitors, they have recourse to murder to preserve it.

" *Secondly*, At the Society Islands, where the same manners and customs prevail, and which have been less frequently visited by Europeans, infant murder is not so often practised; consequently these islands are more populous, although they appear at present to labour under disease in the same degree as Otaheite. It must, therefore, be concluded, that the frequent visits of ships tend to decrease their numbers, not only by inducing a scarcity of food, but by striking at the very root of population.

" War, undoubtedly, must be supposed to have considerable influence on their declension. In that which occurred in the interval between our voyages, it was said that five or six hundred perished, but these were chiefly old men and women; and this was the only war of any consequence since the arrival of the missionaries. There is also reason to believe that it was more frequent formerly than of late years, as the Bounty's mutineers and other deserters strengthened the authority of the present reigning family to a degree never before enjoyed by any chief. Therefore, war cannot be admitted to have had much influence in the present deficiency of numbers.

" The custom of offering human sacrifices we have no reason to believe to have been more freqnent since their discovery. Probably the number offered will amount to twenty in a year, and, as they are always males, this cannot have a very powerful operation.

Polygamy may also have some effect; but as this custom must be supposed always to have existed, it cannot be considered to have had contributed to present depopulation.

Articles 4.—*Report of the Physical and Mathematical Class of the Institute, upon the Question, Are those Manufactures which emit a disagreeable Smell prejudicial to Health?*

THIS paper will be found among our communications. The Editors have thought it right to add some observations of their own. These may have been necessary in Edinburgh, but in London the law of nuisances is so well defined by frequent decisions, that it is unnecessary to do more than hint the general outlines of it.

All inconveniences are cognizable by the justices, with an appeal to the quarter sessions. All unhealthy occupations are removeable. In considering such as are inconvenient by the smell they occasion, or any other cause, the first consideration is, whether the town has has extended to the nuisance, or the nuisance been erected in an inhabited part? In the former case, it is judged unreasonable to dispossess the first occupier. In the latter it is considered, whether the erection, though at first in an inhabited part, was raised without opposition of the neighbourhood, and for how long it continued so; and whether the present possessor gave a consideration on the presumption from former sufferance, that he might carry on his trade without interruption.

Article 5.—*History of the Guinea Worm, and of the Method of Cure employed by the Hindoos.*

THIS is an useful paper, and we sincerely hope that the author, who is not a medical man, has not been deceived by spontaneous cures. He remarks in the early part of the paper, that he administered his remedy almost always with success. "I say," continues he, "*almost always*; because in two or three cases it failed of producing its effect; the person who took it a first time refusing to take it again, which is often necessary when the disease is inveterate. However obstinate the disease may be, the remedy when taken a second time will carry it off, by forcing the worm to come out, if it be already formed, or by preventing its formation, if taken at an early stage of the disease."

A description of the disease follows, and of the worm also, as well as the supposed source of both. By the author's account, the probability would seem that it is imbibed with the common water of the country, and finds its way from the stomach to the extremities; but of this he is too cautious to speak decidedly.

"But whatever may be the cause or origin of the disease," continues Mr. Dubois, "it will be enough for me if the remedy I propose proves a specific for its cure; and I will now relate an instance which, independently of experience, would prove its efficacy. A long time before I was acquainted with this remedy, conversing with the natives on the disease, it was many times remarked that the Bramins were never or very rarely affected with it, although living in villages where the other inhabitants were constantly visited by it every year; but I never paid any attention to such a remark, considering it as one of those idle assertions so common amongst the natives, for which no apparent cause can be alleged, excepting in this case, to attribute that exemption as a privilege exclusively attached to the sacred character of that order of men. Reflecting, however, on that remark, after being acquainted with the remedy, I was led to suppose that this exemption of the Bramins from the disease must be attributed to the constant and daily use which they make of the *assafœtida*, or *peroongahyam*, as one of the principal ingredients in their food, which is also the principal ingredient in the remedy."

The paper concludes with a very sensible letter from Dr. Anderson, who, though less sanguine in the virtues of the remedy, pays a proper respect to this, as to every other suggestion, from whatever quarter.

Article 8.—*Case of Sphacelated Hernia, with Observations.* By GEORGE KELLY, Fellow of the Royal College of Surgeons, Edinburgh.

THIS paper contains some good cases of strangulated hernia which have ended fortunately, the natural passage of the fæces having been restored after an artificial anus had been for a time established. We believe such cases are not so rare as the writers suppose; but till the means of medical communication became so easy as at present, men were less anxious to inform the public of the resources of nature, than of the success of their own operations.

Article 9.—*Case of Tic Doloreuse.* By GEORGE KITSON, Member of the Royal College of Surgeons, London.

IN this instance the pain was situated over the orbit of the left eye, immediately in the situation of the frontal nerve. This last was divided by an incision about an inch in length, directly above the eyebrow down to the bone. A spasm succeeded the operation, but from that time the patient has remained free. The wound healed by the first intent.

We would suggest, whether the success of this operation may not depend on the certainty of dividing the nerve and every collateral branch, which may have an immediate communication. In this case there is the fairest proof of such complete division of the nerve, as the skin of the forehead, in which the divided nerve is distributed, is now deprived of sensation. The operation was more simple than in the more usual place near the zygoma. In these cases we suspect the want of success may arise from some small branches of the nerves below the bony process being left undivided.

Article 10.—*Account of Dr.* GALL'S *Discoveries regarding the Structure of the Brain.* By T. ROSENMULLER, Professor of Anatomy at Leipsic.

Article 11.—*The Enquirer*, No. 6. *On Herpes.*

EVERY practitioner is so sensible of the difficulty of distinguishing, still more of describing, cutaneous diseases, that we are all willing to give as much credit as possible to those who will undertake such a task. Perhaps, as in some other instances, one reason for this general candour may be, that, conscious of our incapacity to satisfy our own minds, we are glad of any authority under which we can shelter ourselves.

The present author attempts only three divisions of cutaneous diseases: papulous eruptions, which, without any discharge, terminate in scurf; eruptions which, though not prone to suppuration, discharge a serous fluid, and terminate in scurf and scales; and pustulous eruptions, which suppurate and terminate in crusts or scabs. Herpes the author confines to those of the second division. "The term herpes," says he, "should be confined to those clusters clusters of minute *pustules* which do not end in suppuration, but discharge a serous fluid and terminate in scurf; sometimes in crusts. It will thus be separated from the simple papulous eruption on the one hand, and from pustular ulcerating affections on the other."

There is much appearance of accuracy in this statement, and it is far from our intention to discourage the endeavours of any one who will undertake so intricate a subject. But we wish every writer, who attempts correctness, to be first mindful of the importance of words: we are not pleased at meeting with the definition of *pustules* without *pus*, or *suppuration.* Perhaps we may be accused of unnecessary nicety in this remark, but we have too good an opinion of the Enquirer to think he will suspect us of any wanton severity.

The history given of herpes is too confined; it is, however, very correct of one species, excepting that, as far as our observations go, the patients are seldom in complete health. Towards evening a slight hectic comes on, during the exacerbation of itching, and sometimes pain in the parts affected is perceivable.

We were much surprized to find the author assert that this disease is not contagious. In cottages it is often found in the whole family, and will sometimes assume different appearances in different subjects, insomuch that nothing but the frequency of the occurrence would convince us that it is the same disease. Had our author witnessed these varieties, we think he would have marked some of them in his description of the disease.

The paper concludes with some rational curative plans, and a proper reflection on the danger that is unnecessarily apprehended from the application of local remedies.

Under the third department of this Number, or Medical Intelligence, we have an account of the Medical Topography of Berlin, with the public institutions for the cultivation of medicine and for the relief of the sick, and a list of the diseases for the year 1804: such records as these are always useful.—Dr. R. Pearson's mode of treating the hooping cough, which contains nothing new to our readers in the South; the custom of giving repeated emetics being happily exploded for some years past.—Mr. Wilkinson's letters on his want of success in repeating the galvanic experiments of Pacchioni and Mr. Peele, without producing a similar result.—A repetition of the old questions from the Cancer Institution, from whom every medical man has been impatiently expecting some report: [Are these gentlemen to be for ever questioning without informing? and is all their own and other peoples knowledge of that disease to remain for ever an arcanum, confined to their own archives?]—The names of the gentlemen who received 'Doctor's degrees at the last SENATUS ACADEMICUS, with the titles of their Theses; and the Quarterly Report from the Cary Street Dispensary.

*The Domestic Medical Guide in Two Parts. Part I. The Family Dispensatory, or, a complete Companion to the Family Medicine Chest, &c. Part II. The Modern Domestic Medicine, comprehending the most approved Methods of treating and obviating the different Diseases that assail the Human Frame; with the most important Information relative to the Cure of those Chronic Diseases which have been generally considered Incurable. Third Edition considerably enlarged and corrected.* By Richard Reece, M. D. late of St. Bartholomew's, and the General Infirmary at Hereford, Fellow of the Royal College of Surgeons in London, Author of the Medical and Chirurgical Pharmacopœia, &c. 8vo. 1805.

Whenever doubts are entertained as to the necessity of medicine and physicians, one answer is always at hand, and is perhaps the best as well as the safest that we can make. If no one were to study the nature of diseases and remedies, every one would be alike unable to propose the mode of cure, and more mischief would be done by those who are altogether ignorant, than by such whose education and experience, enable them to judge of the resources of nature, and of the properties of medicine. On this account, we are never averse to books of this kind, when they are written with caution, and pretend only to instruct families till they can procure the best advice. By these means they lessen the impatience of the ignorant, and prevent the mischief which might be done by the forward and conceited.

While we make these allowances for works not strictly correct, or to speak more technically, not perfectly regular; we cannot approve of that affectation of superior knowledge, which will always have a certain degree of effect with those who are not competent to judge. Thus, we think many of the authors *rubs* on Dr. Buchan might have been spared. It is true, that writer proposes opening the wind-pipe in cases, where no means of removing a substance from the gullet prove successful. To this, our author says, "What advantage can possibly be derived from making an opening in the wind-pipe, to remove a substance lodged in a different tube, must puzzle an anatomist to conjecture. Should an apothecary, not conversant in anatomy, attempt to relieve a person in great agony and apparent danger, by following the author's advice, and after making an incision in the wind-pipe, and not finding the obstructing body there, should be induced to cut into the gullet, the life of the patient would be inevitably destroyed."

Now we thought both these doctors had written for families, who could not procure an apothecary in time. For Dr. R. should recollect, that apothecaries are now generally like himself, Fellows of the College of Surgeons in London, or educated in the same manner. If a gentleman of this description should arrive, though, like Dr. Reece, he should submit to keep an open shop, yet the probability is, that he would dissect carefully for the gullet. But before before his arrival, the patient might die, for want of expanding the lungs from the mechanical pressure of the trachea by the substance lodged in the gullet. We have not seen what Dr. Buchan says on this subject, but certainly all this is to be expected; and forlorn as the chance may be, it probably is the only one for saving the patient in such an emergency.

Under the article phthisis pulmonalis, the author has availed himself of that division suggested by Dr. Adams, in his paper on the climate of Madeira, contained in the fifth volume of our Journal. We wish Dr. Reece had chosen a better term than adhesu (*ahesu* must be an error of the press), a very unclassical word, and by no means explicit, as it should rather be appropriated to common adhesion with the pleura when applied to the lungs.

---

*Exposition on the Inoculation of the Small Pox, and of the Cow Pock.* By JOHN COAKLEY LETTSOM, M. D. LL. D. and Member of several Academies and Literary Societies. Second Edition, 1806.

THIS is a well meant effusion on the advantages of a practice, which we believe, would have been more general, if its friends had been less zealous. But the same warmth of temper which leads to one system, is equally impetuous in another. Thus, the Ephesian matron, who could exist no where but by her dead husband's corpse, was the readiest at receiving the addresses of a living suitor.

This allusion is not entirely out of point, when we consider how great an advocate Dr. Lettsom was for small-pox inoculation, without even those cautions which Baron Dimsdale's good sense and moderation thought it right to propose; yet the Baron never condescended to call names: he conceived that Drs. Wilkinson, Lettsom, and others, had very good intentions, though they might be somewhat mistaken.

We are aware it will be said, that when Dr. Lettsom supported his friend Dr. Wilkinson, and others, the cow-pox was unknown. But what would this prove? That all prudent people have "it providentially *now* in their power to prevent the bad consequences which result from the small pox, by the medium of the cow pock." Unhappy the fate of those who formerly "were without this protection, when Dr. Wilkinson tells us, that the physicians to the society inoculate in narrow streets, in little courts, and in the midst of those who have not had the disease; and even on ground floors, where a number of children continue to play during the course of their illness; in short, where the intercourse between the well and the sick is unavoidable; and without taking the least care to prevent the infection from spreading." Such was the practice of one with whom Dr. Lettsom "entertained a similar opinion." What must have become of the unhappy manes of poor Dr. Wilkinson, who, there is reason to fear, died in his sins; how much we rejoice, that our old friend Lettsom has lived to repent!

---

*An Encyclopædia of Surgery, Medicine, Midwifery, Physiology, Pathology, Anatomy, Chemistry, &c. &c. &c. to which is added, an abridged Translation of Cullen's Nosology.* By JOHN JAMES WALL, Surgeon, 1806.

THIS is a handy little volume, and contains more useful references than most others of a much larger size.

---

*An Examination into the Principles of what is commonly called the Brunonian System introductory to a Series of Aphorisms upon Life and Mind, Health and Disease, with an Attempt to form a more simple and Philosophical Arrangement of Diseases, upon the present State of our Knowledge of the Animal Economy.* By THOMAS HARRISON, *Member of the Royal College of Surgeons in London, &c. &c.*

THE only advantage we ever could find in the Brunonian system has been, that every one moulded it in some measure to his own opinion. We mean not John's own theory, for that was stubborn, and to that he died a martyr; but we mean the manner in which it has been at different times modelled; however, one thing is very remarkable, that no person to whom the public has looked up as possessing superior talent, has given himself any concern about it. The writer before us, is certainly a man of no mean abilities or inconsiderable erudition, and as such we hope to see him employed in some original work more worthy of himself than the subject he has now selected.

The author informs us that he felt particularly disposed to this task, by perusing Dr. Garnet's Lectures on Zoonomia.

"In pursuance of this intention," says he, "I shall consider the Brunonian System in four points of view; and endeavour to prove, in the first place,

"That its principles are not founded upon the true laws of the animal economy;

"Next, That they are contradictory and inconsistent in themselves;

"Thirdly, That they are not sufficiently general in their application to diseases; and,

"Finally, That they may lead to dangerous errors in practice.

"As it has been said by Dr. Garnett, that Brown did not clearly understand his own system; in order to meet the question as fairly and fully as the subject requires, I shall discuss each particular head of examination with a reference, as far as is necessary, to the sentiments of both these gentlemen, as delivered in their particular works; and as the favourers of the Brunonian System have ever been particularly desirous of making it appear that his theory is is founded upon the principles of the Newtonian Philosophy, I shall require no other ground for my reasoning than these principles, taken in their fullest extent; and in particular Sir Isaac Newton's first rule of philosophising, and the law of gravitation, mentioned by Dr. Garnett as having been particularly kept in view by Dr. Brown in all his deductions.

"The first rule of Philosophising is, that no other causes of natural things ought to be admitted but such as are true in fact, and sufficient to explain the phenomena. This is agreeable to those received maxims, that Nature does nothing in vain, and that it is in vain to make use of more things where fewer will do as well; for Nature always takes the best and wisest course, that is, the most simple and free from superfluity. Gravitation, according to Sir Isaac Newton's Theory of the Universe, is that power by which all heavy bodies near the earth tend to its centre, with a force proportionate to their quantity of matter: by this law the moon also tends to the centre of the earth, and the waters of the sea to the centre of the moon; and by the equal and uniform action of the same power, the earth and moon, planets and comets, tend towards the sun and towards each other. In addition to these laws, the latter of which is a simple, beautiful, and sublime illustration of the former, I shall beg leave to add an axiom more intimately connected with the subject; an axiom that may be made as clear to our senses as any in Euclid's Elements of Mathematics, viz.

"That as life is only known in an organized body by its effects, and as the cause of an effect must be different from that effect, the effects of life cannot be life.

"The whole of the Brunonian Theory appears to be founded upon the application of the three words, incitabilitas, incitatio, and stimulus. Incitabilitas is by Dr. Garnett translated excitability, and incitatio by the English word excitement. These terms are so much in the vein of some of our late medical and chemical writers, that I hope the reader will pardon me a short digression in this place, while I express a sentiment of reprobation against the too prevalent practice of coining new words upon the most frivolous pretences. It appears to me not only pedantic and affected, and very much beneath an author who has any the most distant pretension to real knowledge, but it is even in its consequences highly injurious to the diffusion of science. Whenever I find a writer attached to this sort of coining, I immediately suspect, and generally find my suspicions well founded, that his ideas of his subject are not sufficiently clear, and that, by the intervention of new terms, not well defined, he endeavours to throw a mist before the eyes of his reader, that he may not discern his imperfections.

"If a word cannot be found in the language in which an author writes that will fully express his meaning, he will generally find one, which by adding to, or taking a little from the common received sense, will very well answer his purpose; the change will be easily carried, with the general received acceptation of the word, into the reader's memory, and his deductions will become clear in proportion. In the case, alone, of such a word not being to be found in a language should the introduction of a new one be admitted.

"I trust this short digression will be found not altogether foreign to the subject, as almost the whole of Dr. Brown's new terms, and his old words with new meanings attached to them, will be found deserving of this censure to the fullest extent.

"Brown, in his general acceptation of the word excitability, appears evidentally to mean the vital and mental principle or life and mind. After stating the difference between living and inanimate matter, and the powers which he supposes to be productive of that difference, he proceeds to specify the vital and mental actions themselves, and their absolute necessity to life. He then says, 'Proprietas per quam utræque agunt incitabilitas dicenda.' Agreeably to this definition, I shall therefore wish the words excitability, vital principle, or life, wherever they occur in the course of this essay, except where a contrary sense is particularly attributed to them, to be considered synonimous, and that by them I mean to express the power which produces all the vital and mental actions. Dr. Garnett says, we may call this property, with Haller, irritability; or with Brown, excitability; or we may use the words vital principle. The term excitability he prefers, as perhaps least liable to exception; for what reason I cannot perceive, unless from his particular partiality to its author.

"Excitement, according to Brown's use of the word, is the effect of excitability, or the vital principle in producing the vital actions: 'Potestatum incitantium (incitabilitatem) communis effectus sensus, motus, mentis actio, et animi adfectus sunt.' And again, 'Potestatem incitantium, incitabilitatem agentium, effectus incitatio nuncupandus.' By stimuli or exciting powers are meant certain things, most commonly external, that are supposed to act upon the excitability, as above stated, and produce excitement, or the vital actions. Those of the greatest consequence are heat, food, air, the blood, and secreted humours, &c.

"As there appears some difference of opinion between Brown and his disciple respecting the application of the words excitability and excitement, and as much confusion of thought seems to run through this part of the Brunonian Theory, it will be highly necessary to endeavour fully to develope their meaning in this place; since the truth or fallacy of every conclusion formed by them rests upon the application of the above three terms.

"Dr. Garnett, notwithstanding his definition of excitability as above, seems in all his subsequent reasoning to have understood life to be placed in excitement. It cannot be that by the vital principle he means the nervous influence acting upon the nervous system, and producing the actions of life; for in this case his conclusion would be glaringly erroneous; because the nervous influence, or or call it what you will, must still be acted upon by excitability, which would be the moving power, and the vital actions the mere effects of its influence. On the other hand, if we take excitability according to his own definition of the term, he makes the exciting powers act upon life to produce life. Now Brown is here perfectly consistent: he never calls the vital actions life; but says, what is the fact, that they are the only means of discrimination between life and death. By excitability, Brown uniformly means life itself, at least as far as can be collected from the whole tenor of his work: it is not only the mover of the sensorial power, but of all the vital and even the mental actions, 'sensus, motus, mentis actio, et animi.' The nervous influence, whatever it is, can only exert its powers upon the nervous system. The vital principle pervades every vital and mental action: perception, thought, sensation, circulation, respiration, motion."

We have made this copious extract, because we really know not how to compress the author's meaning; for he has, with much candour, given something like a meaning where we could discover none, embodied a fleeting shadow, and we despair of expressing ourselves with so much point and fluency as he every where shows. It may be from a want of patience, or from an impetuosity which might better become younger people; but whenever we discover a meaning about Brown or his followers, it amounts to so little, that we are never satisfied we can be right, or never satisfied that they are right. This discouragement has pervaded our every enquiry for the last twenty years that the doctrine has been afloat, and during that period we are all become too old, we fear, to mend. We were therefore much pleased to see a compact statement of the doctrine which the author felt himself obliged to make before he undertook to confute it, and we recommend the work to such of our readers as wish for information on a subject at one time so popular.

